# The mGluR6 ligand-binding domain, but not the C-terminal domain, is required for synaptic localization in retinal ON-bipolar cells

**DOI:** 10.1016/j.jbc.2021.101418

**Published:** 2021-11-15

**Authors:** Melina A. Agosto, Abiodun Adefola R. Adeosun, Nitin Kumar, Theodore G. Wensel

**Affiliations:** 1 Verna and Marrs McLean Department of Biochemistry and Molecular Biology, Baylor College of Medicine, Houston, Texas, USA; 2 Pharmacology and Chemical Biology Graduate Program, Baylor College of Medicine, Houston, Texas, USA

**Keywords:** retina, synapse, metabotropic glutamate receptor (mGluR), photoreceptor, trafficking, BC, bipolar cell, CSNB, congenital stationary night blindness, CT, C-terminal tail, LBD, ligand-binding domain, OPL, outer plexiform layer, TRPM1, transient receptor potential melastatin

## Abstract

Signals from retinal photoreceptors are processed in two parallel channels—the ON channel responds to light increments, while the OFF channel responds to light decrements. The ON pathway is mediated by ON type bipolar cells (BCs), which receive glutamatergic synaptic input from photoreceptors *via* a G-protein-coupled receptor signaling cascade. The metabotropic glutamate receptor mGluR6 is located at the dendritic tips of all ON-BCs and is required for synaptic transmission. Thus, it is critically important for delivery of information from photoreceptors into the ON pathway. In addition to detecting glutamate, mGluR6 participates in interactions with other postsynaptic proteins, as well as trans-synaptic interactions with presynaptic ELFN proteins. Mechanisms of mGluR6 synaptic targeting and functional interaction with other synaptic proteins are unknown. Here, we show that multiple regions in the mGluR6 ligand-binding domain are necessary for both synaptic localization in BCs and ELFN1 binding *in vitro*. However, these regions were not required for plasma membrane localization in heterologous cells, indicating that secretory trafficking and synaptic localization are controlled by different mechanisms. In contrast, the mGluR6 C-terminus was dispensable for synaptic localization. In mGluR6 null mice, localization of the postsynaptic channel protein TRPM1 was compromised. Introducing WT mGluR6 rescued TRPM1 localization, while a C-terminal deletion mutant had significantly reduced rescue ability. We propose a model in which trans-synaptic ELFN1 binding is necessary for mGluR6 postsynaptic localization, whereas the C-terminus has a role in mediating TRPM1 trafficking. These findings reveal different sequence determinants of the multifunctional roles of mGluR6 in ON-BCs.

Vision begins in the retina with rod and cone photoreceptor cells, which are highly specialized for detection of photons. Information is then relayed and processed through circuits involving different types of retinal neurons connected by chemical and electrical synapses, before eventual delivery to the brain. In the outer plexiform layer (OPL) of the retina, photoreceptors form chemical synapses with different types of bipolar cells (BCs). In the dark, photoreceptors release the neurotransmitter glutamate into the synapse; light onset leads to suppression of glutamate release. At the OPL, visual information is divided into two channels (1). The ON channel is mediated by ON-BCs, in which the metabotropic glutamate receptor mGluR6 is negatively coupled to a cation channel, resulting in depolarization in response to light. Meanwhile, the OFF channel is mediated by OFF-BCs, which express ionotropic glutamate receptors, resulting in hyperpolarization in response to light. Cone photoreceptors, which function in bright light, form synapses with multiple types of both cone-ON and cone-OFF BCs. Rod photoreceptors, which function in dim light, synapse instead with rod BCs, which are of the ON type (2). Thus, the ON pathway is essential for dim light vision.

As the glutamate detector in both rod BCs and cone-ON BCs, mGluR6 is necessary for receiving photoreceptor input into the ON pathway (3, 4, 5, 6). mGluR6 is a class C GPCR that is coupled to G_αo_ in BCs (7, 8, 9); G_αo_ and/or G_βγ_ regulate a cation channel that requires the transient receptor potential melastatin (TRPM1) protein (10, 11, 12, 13, 14, 15). In addition to its role in detecting glutamate, mGluR6 also plays a role in mediating synaptic localization of TRPM1 (16, 17). Mutations in the *GRM6* gene, which encodes mGluR6, are associated with congenital stationary night blindness (CSNB) in humans, and both human patients and mouse models in which mGluR6 is ablated exhibit a lack of b-wave in electroretinograms, indicating ON-BC disfunction (3, 18, 19, 20).

Like other mGluR family members, mGluR6 has a large extracellular venus flytrap ligand-binding domain (LBD) that is connected to the transmembrane domain by a cysteine-rich region, and a short C-terminal tail (CT) that is located in the cytoplasm. The extracellular domain of mGluR6 participates in trans-synaptic interactions with the leucine-rich repeat proteins ELFN1 and ELFN2, expressed presynaptically in rods and cones respectively (21, 22). Knockout of ELFN1 results in loss of mGluR6 synaptic localization in rod BCs and malformation of the rod synapse ultrastructure (21). Knockout of ELFN2 results in compensation by expression of ELFN1 in cones, but double knockout of ELFN1 and ELFN2 results in similar loss of mGluR6 at cone-ON BC dendritic tips except without apparent structural defects (22).

The mechanisms by which mGluR6 is directed to the BC dendrites, and ultimately the postsynaptic membrane, are unknown. In this study, we show that multiple regions of the mGluR6 LBD are essential for dendritic tip localization in retinal BCs, as well as for ELFN1 binding, but surprisingly are dispensable for plasma membrane trafficking in heterologous cells. We further show that the cytoplasmic CT is not required for dendritic tip localization, even in the absence of endogenous mGluR6, though it may play a role in TRPM1 trafficking.

## Results

### The mGluR6 ligand-binding domain, but not the C-terminal tail, is necessary for dendritic tip localization in ON-BCs

**Figure 1:**
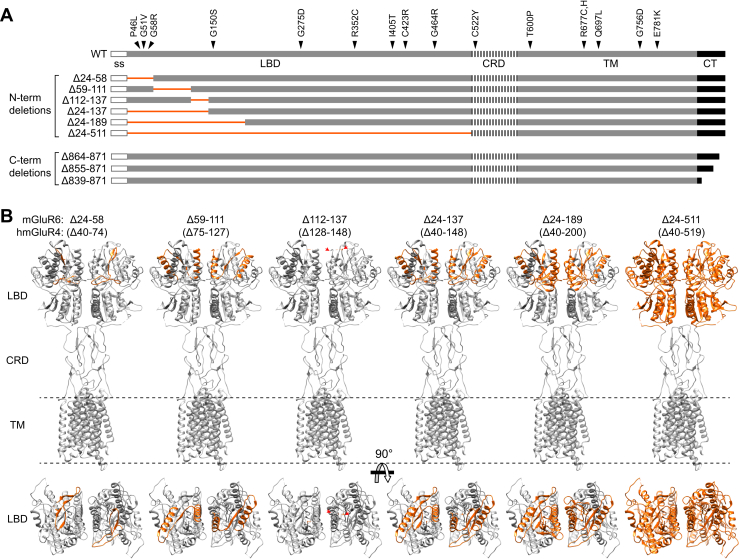
**Deletion mutants.***A*, diagram of murine mGluR6 deletion mutants. Human CSNB missense mutations (36, 65, 66), with *arrows* indicating homologous positions in murine mGluR6, are shown. *B*, deleted regions shown in the structure of human mGluR4 (PDB 7E9H (67)) (homologous positions in mGluR4 are indicated in *parentheses*) in side views (*top*) and top views (LBD only, *bottom*). The mGluR6 region 112 to 137 has very low sequence similarity to mGluR4, and residues 128 to 147 in the homologous region are not visible in the mGluR4 structure (*red arrows*). The cytoplasmic CT domain is also absent in the mGluR4 structure. CRD, cysteine-rich domain; CT, C-terminal domain; LBD, ligand-binding domain; ss, signal sequence; TM, transmembrane domain.

**Figure 2:**
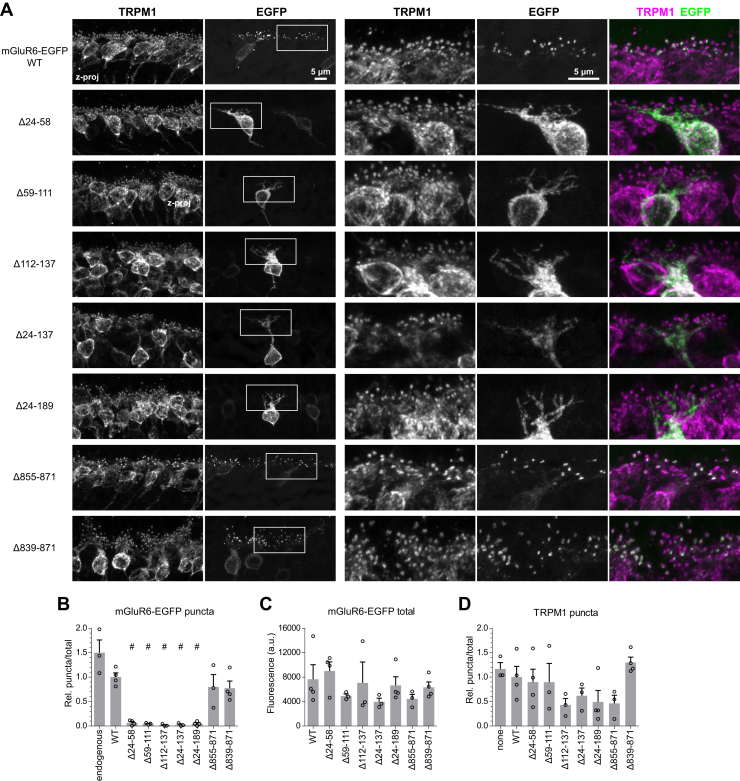
**Localization of mGluR6 mutants in WT retina.***A*, images of WT CD1 retinas electroporated with WT or mutant mGluR6-EGFP and immunostained with TRPM1 antibody. *Boxes* show location of higher magnification views on the *right*. *B*, quantification of mGluR6-EGFP OPL puncta, shown relative to total EGFP and normalized to WT. *Dots* represent means of at least three images each from biological replicates and error bars show means ± SEM. Quantification of endogenous mGluR6 labeled with antibody clone 366 (see Fig. 7) is included for comparison. *C*, quantification of total EGFP fluorescence in the same images used for (*B*). *D*, quantification of TRPM1 OPL puncta, shown relative to total TRPM1 staining and normalized to WT, in the same images used in (*B*). ^#^*p* ≤ 0.001, one-way ANOVA with Dunnett’s post-test to compare all mutants to WT.

To investigate regions of mGluR6 involved in trafficking and synapse localization, we constructed deletion mutants in the N-terminal LBD and the C-terminal cytoplasmic tail (CT) (Fig. 1). mGluR6 WT and mutant constructs with C-terminal EGFP fusions were expressed in ON-BCs in WT CD1 mice by *in vivo* electroporation (23). WT mGluR6-EGFP colocalized with dendritic tip puncta in the OPL, as expected (24). CT deletions, including removal of nearly the entire region following the last transmembrane helix, did not affect dendritic tip localization at the resolution of confocal microscopy. In contrast, all of the LBD deletions were mislocalized, with no discernable dendritic tip accumulation (Fig. 2*A*). Total GFP fluorescence in the imaged fields was similar for all mutants (Fig. 2*C*). OPL puncta intensity was quantified in both EGFP and TRPM1 channels (Fig. S1). LBD mutants had drastically reduced OPL puncta intensity (Fig. 2*B*), while TRPM1 was similar for all mutants, showing that dendritic tips were present and that OPL regions were appropriately defined for quantification (Fig. 2*D*).

**Figure 3:**
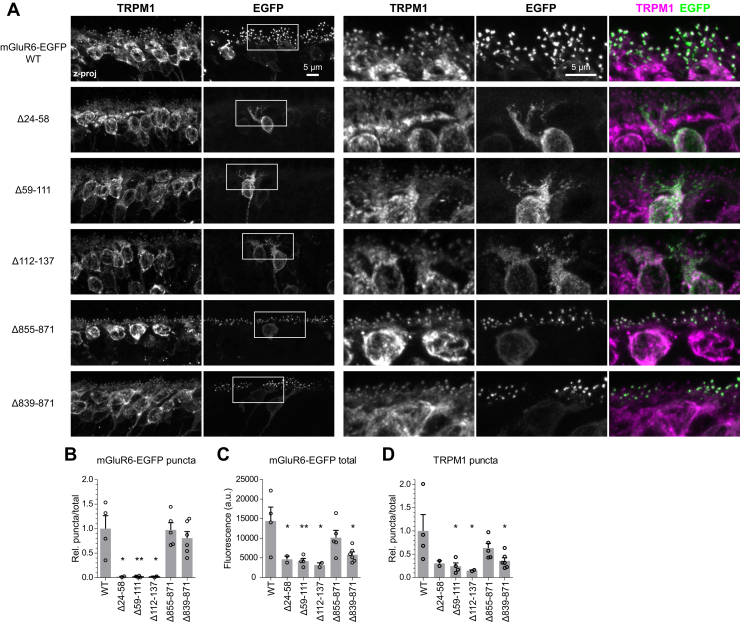
**Localization of mGluR6 mutants in nob3 retina.***A*, images of CD1/nob3 retinas electroporated with WT or mutant mGluR6-EGFP and immunostained with TRPM1 antibody. *Boxes* show location of higher magnification views on the *right*. *B*–*D*, quantification of mGluR6-EGFP OPL puncta, total EGFP, and TRPM1 OPL puncta, as described in the Figure 2 legend. *Dots* represent biological replicates. ∗*p* ≤ 0.05; ∗∗*p* ≤ 0.01, one-way ANOVA with Dunnett’s posttest to compare all mutants to WT.

Since other mGluR family members are known to form homodimers (25), we asked whether the presence of WT endogenous mGluR6 was able to affect the localization of mutants introduced by electroporation. *Grm6*^*nob3*^ mice (hereafter nob3) have a naturally occuring *Grm6* mutation in the intron between exons 1 and 2, leading to insertion of a spurious exon containing a premature stop codon; no detectable mGluR6 protein is produced (20). The localization of mGluR6 mutants in these mice was indistinguishable from that observed in WT CD1 mice (Fig. 3, *A* and *B*). These results indicate that correct localization of CT mutants is not mediated by dimerization with a WT protein. Further, they suggest that LBD mutants either cannot form heterodimers with WT mGluR6 or that dimerization is not sufficient to rescue localization.

### The mGluR6 CT is involved in mediating TRPM1 dendritic tip localization

**Figure 4:**
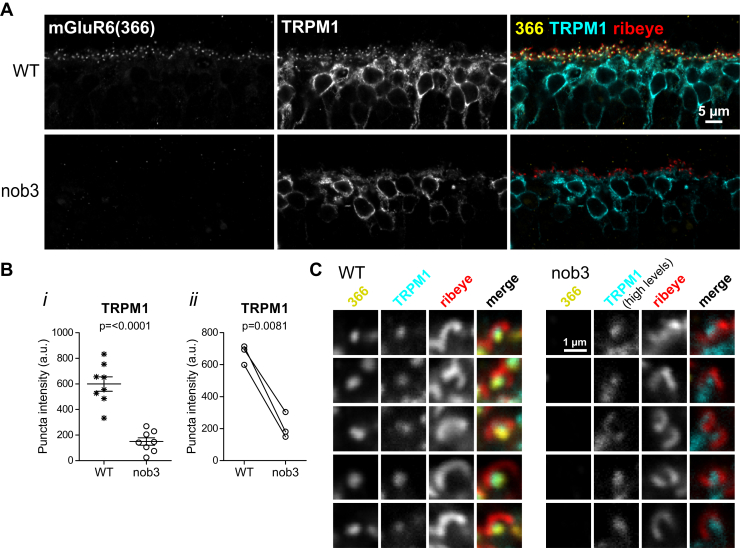
**Reduced TRPM1 puncta accumulation in CD1/nob3 mice.***A*, retina sections from WT CD1 or CD1/nob3 mice were immunostained with antibodies for mGluR6 (clone 366), TRPM1, and ribeye. *B*, comparison of OPL puncta intensity in WT CD1 and CD1/nob3 retina sections. i, example quantification of technical replicate images from one experiment. Genotypes were compared with two-tailed unpaired *t*-tests. ii, means of biological replicates from three experiments; for each experiment, nob3 and WT retina sections were labeled on the same slide. Genotypes were compared with a two-tailed paired *t* test. *C*, magnified views from images as shown in (*A*). Brightness of the TRPM1 channel was increased for nob3 images to highlight the presence of residual TRPM1 at dendritic tips.

**Figure 5:**
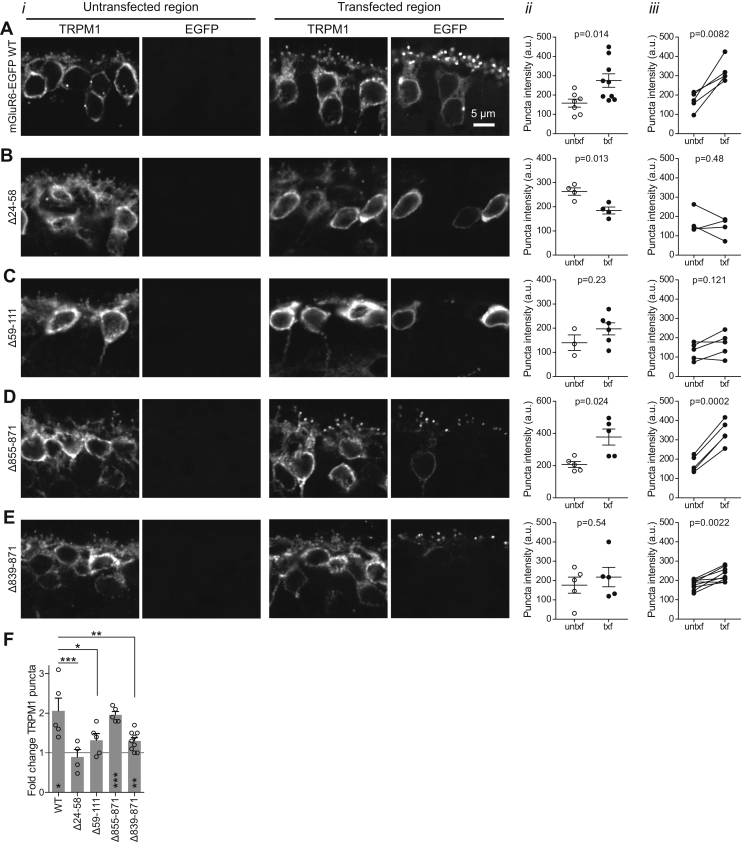
**Rescue of TRPM1 dendritic tip localization by electroporated mGluR6-EGFP.***A*–*E*, i, examples of TRPM1 immunostaining in untransfected and transfected regions of the same retina. ii, example quantification of technical replicate images from one retina. Untransfected (untxf) and transfected (txf) regions were compared with two-tailed unpaired *t*-tests. iii, means of biological replicates were compared with two-tailed paired *t*-tests. *F*, summary of fold changes in TRPM1 puncta intensity in transfected regions. *Asterisks* on the bars indicate one-sample *t* test with theoretical mean of 1, and *asterisks* above the bars indicate comparison with WT using one-way ANOVA and Dunnett’s posttest. ∗*p* ≤ 0.05; ∗∗*p* ≤ 0.01; ∗∗∗*p* ≤ 0.001.

As reported previously for nob3 mice and other mGluR6-ablated strains (16, 17), TRPM1 dendritic tip accumulation was significantly reduced in nob3 mice, compared with WT mice (Fig. 4, *A* and *B*). TRPM1 was not completely absent from dendritic tips however—reduced labeling was still detectable in structures directly apposed to staining for the presynaptic marker, ribeye (Fig. 4*C*). In images of nob3 retinas electroporated with some mGluR6 mutants, TRPM1 OPL puncta intensity was reduced compared with images of nob3 retinas electroporated with WT mGluR6-EGFP (Fig. 3, *A* and *D*), suggesting that mutants may have a differential ability to rescue TRPM1 localization. To examine more carefully whether electroporated mGluR6-EGFP can rescue TRPM1 localization, images of transfected regions (containing EGFP) and untransfected regions (where no EGFP was detectable) were compared (Fig. 5). Overall, WT mGluR6-EGFP and the intermediate CT mutant Δ855–871 mediated significant rescue of TRPM1 OPL puncta intensity (Fig. 5, *A* and *D*), although the technical replicate images from transfected and untransfected regions within one retina (for example, Fig. 5*A*, ii) were not significantly different for every animal (WT: *p* ≤ 0.05 for two of five animals; Δ855–871 *p* ≤ 0.05 for three of five animals) (Fig. S3). The complete CT deletion mutant Δ839–871 also appeared to mediate a smaller, but significant, increase in TRPM1 puncta localization (Fig. 5*E*). However, the images of transfected and untransfected regions were not significantly different for any of the nine animals electroporated with this mutant (Fig. S3; example in Fig. 5*E*, ii), lending uncertainty to the result. The extent of TRPM1 rescue measured by the Δ839–871 mutant was significantly less than that mediated by WT mGluR6 (Fig. 5*F*), suggesting a role for the mGluR6 CT in TRPM1 localization. The LBD mutants Δ24–58 and Δ59–111 failed to rescue TRPM1 localization (Fig. 5, *B* and *C*).

### Ligand-binding domain mutants are mostly intracellular in ON-BC somas

**Figure 6:**
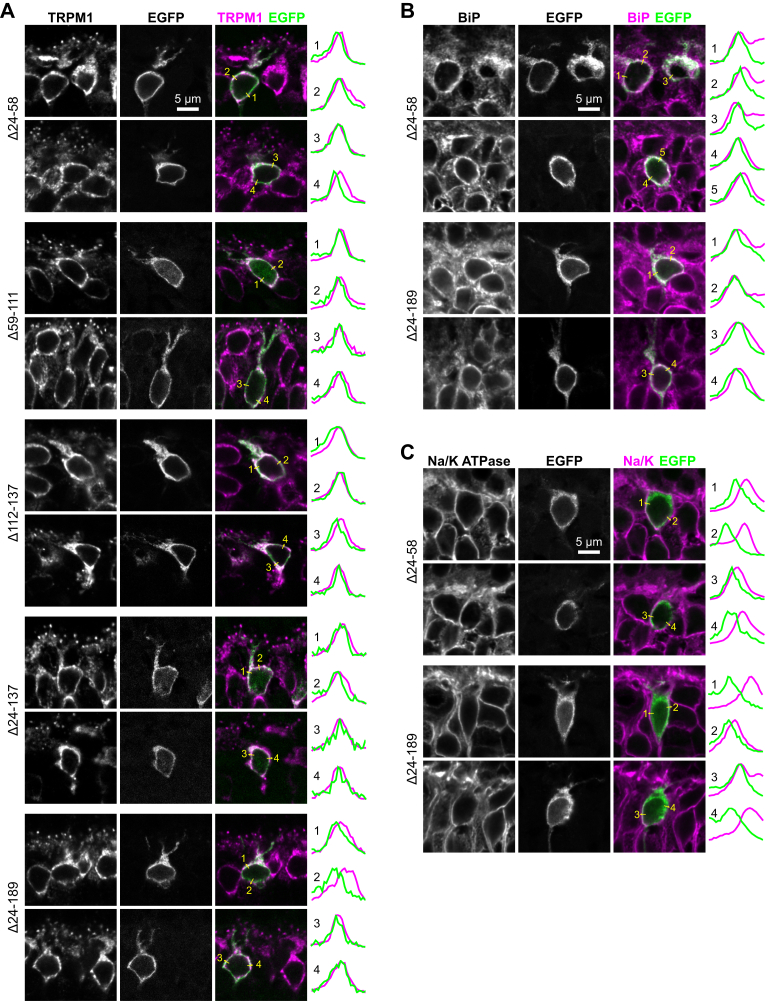
**Intracellular location of mGluR6 LBD mutants.** WT CD1 retinas electroporated with mGluR6-EGFP LBD mutants were immunostained for TRPM1 (*A*), ER marker BiP (*B*), or plasma membrane marker Na/K ATPase (*C*). Intensity profiles are shown for the lines overlaid on the images.

Next, we asked whether the mislocalized mutants are intracellular, or at the plasma membrane but merely not concentrated at dendritic tips. The localization of all LBD mutants in somas was similar to that of somatic TRPM1 (Fig. 6*A*), which was previously shown to be mostly localized to the ER (24). Furthermore, Δ24–58 and Δ24–189 were partially colocalized with the ER marker BiP (Fig. 6*B*), while poorly colocalized with the plasma membrane marker Na/K ATPase (Fig. 6*C*), indicating a primarily intracellular location.

### Novel mGluR6 mAbs reveal endogenous mGluR6 in somas

**Figure 7:**
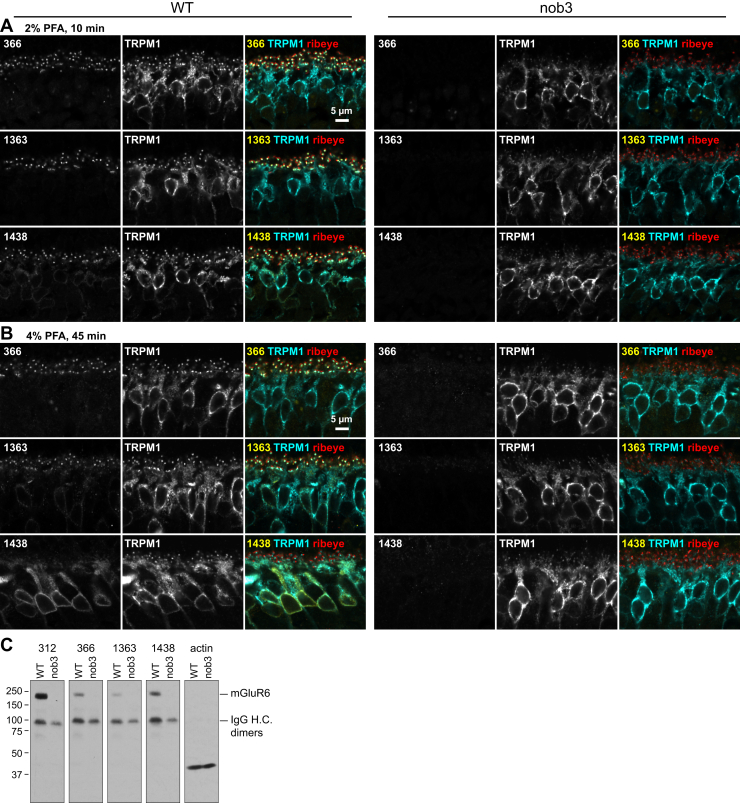
**Specificity and fixation-dependent immunostaining of mGluR6 mAbs.***A* and *B*, WT C57 or nob3 eyes were fixed in 2% PFA for 10 min (*A*) or 4% PFA for 45 min (*B*), and sections were immunostained with an mGluR6 mAb, along with antibodies for TRPM1 and ribeye. *C*, western blots with WT C57 and nob3 retina tissue. The position of endogenous mouse IgG heavy chain dimers, detected with the secondary antibody, is indicated.

**Figure 8:**
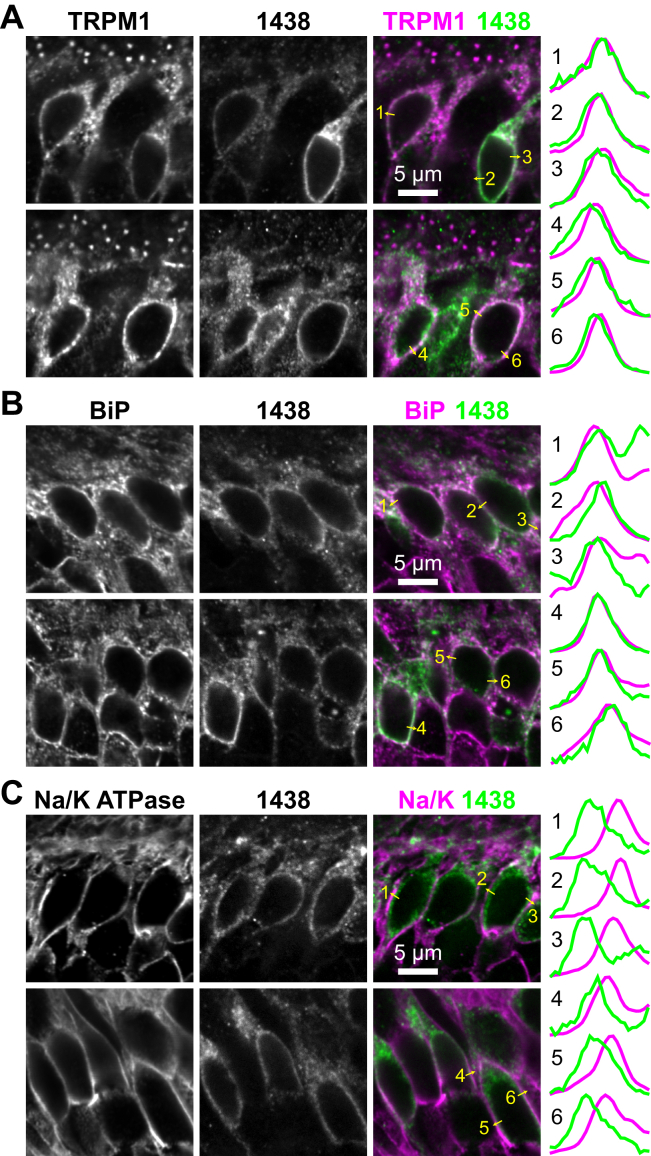
**Intracellular location of somatic mGluR6.** WT C57 retina sections were labeled with mGluR6 mAb 1438 and antibodies against TRPM1 (*A*), ER marker BiP (*B*), or plasma membrane marker Na/K ATPase (*C*). Intensity profiles are shown for the lines overlaid on the images.

To study plasma membrane trafficking without addition of an N-terminal tag, which may affect the results, we characterized a panel of mGluR6 mAbs to identify one with an extracellular epitope. Several clones that detect mGluR6 specifically by immunofluorescence microscopy (IF) and/or immunoblots of retina tissue were identified (Figs. 7 and S4); clones 312 and 366 were previously reported (26, 27). In conditions of mild fixation, all IF-competent clones labeled dendritic tips, as expected (Fig. 7*A*). Interestingly, in harsher fixation conditions, clone 1438 lost the ability to detect dendritic tips and instead labeled BC somas (Fig. 7*B*). Clone 1363 also labeled BC somas, to a lesser extent, in addition to dendritic tips (Fig. 7*B*). Like the dendritic tip labeling, the soma labeling was present in WT, but not nob3, animals, indicating that it is specific for mGluR6. Similar to the mislocalized LBD mutants, endogenous somatic mGluR6 detected with clone 1438 was partially colocalized with TRPM1 and BiP, but not the plasma membrane marker Na/K ATPase (Fig. 8).

**Figure 9:**
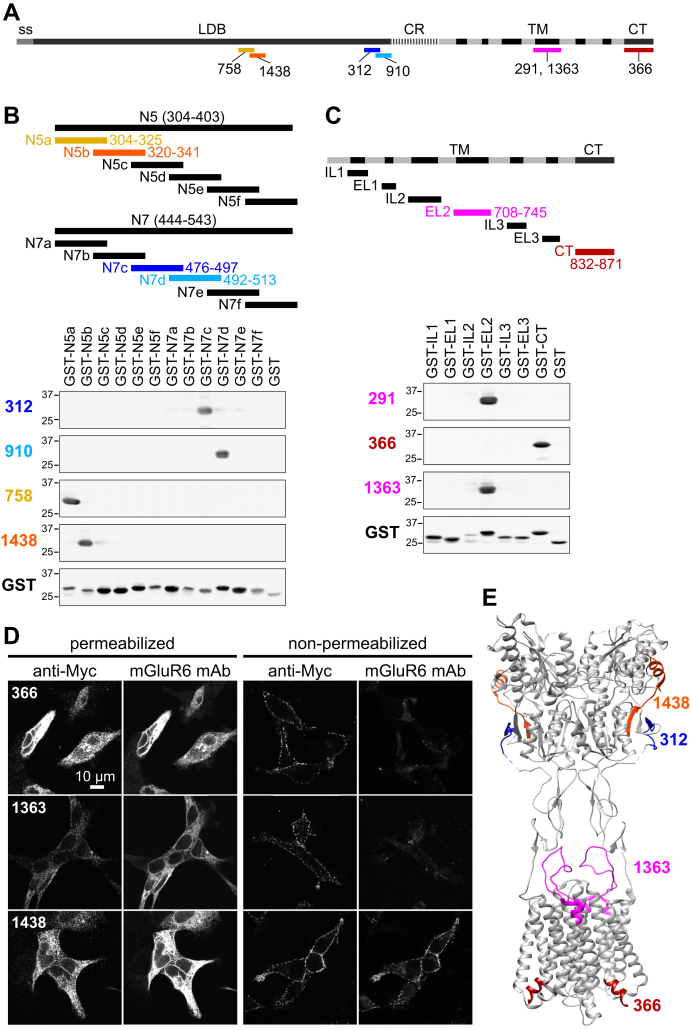
**mGluR6 mAb epitope mapping.***A*, diagram of mGluR6 with mAb epitopes shown. *B* and *C*, following preliminary mapping to segments N5, N7, and TM/CT as shown, western blots were performed with overlapping fragments fused to GST, and mGluR6 or GST antibodies. *D*, HEK cells were transfected with ss-Myc-mGluR6 and labeled in either permeabilizing or nonpermeabilizing conditions with both Myc and mGluR6 antibodies. *E*, structure of mGluR4 (PDB 7E9H) with positions homologous to mGluR6 mAb epitopes shown. Most of the CT epitope is not visible in the structure.

Epitope mapping using western blots of overlapping mGluR6 fragments revealed a variety of epitopes (Fig. 9). Clone 366, which yielded the expected ON-BC dendritic tip labeling, detected an epitope in the CT domain. Of the four clones with LBD epitopes, only 1438 detected mGluR6 in IF of retina tissue (Figs 7 and S4). This clone also labeled plasma membrane mGluR6 in nonpermeabilized transfected HEK cells (Fig. 9*D*). Clone 1363, though mapped to extracellular loop 2, failed to label nonpermeabilized cells. Clone epitopes and properties are summarized in Table S1.

### Ligand-binding domain deletion mutants have enhanced plasma membrane trafficking in heterologous cells

**Figure 10:**
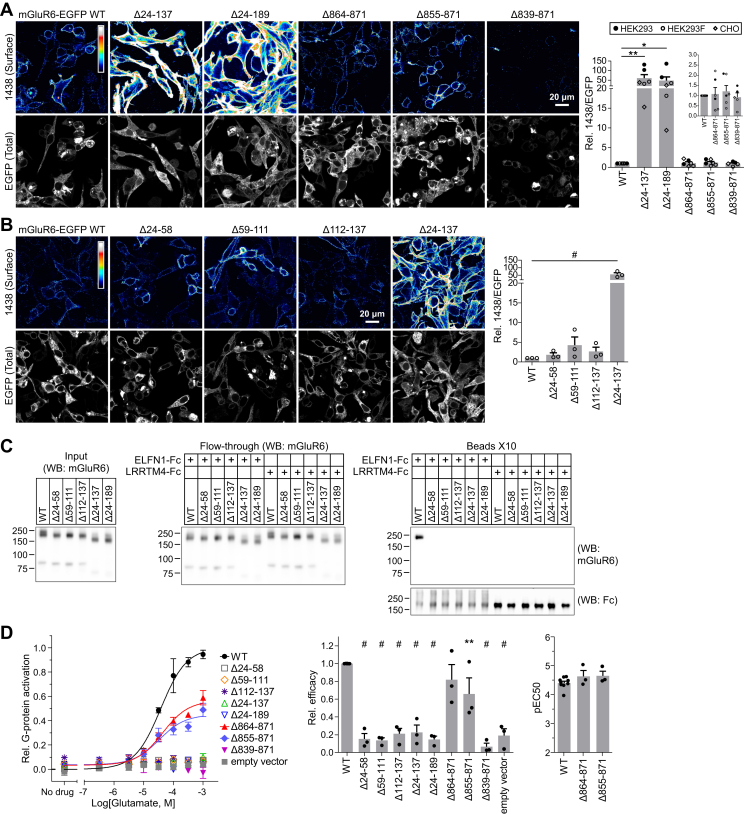
**Surface expression and ELFN1 binding of mGluR6 mutants in heterologous cells.***A* and *B*, HEK293, HEK293F (shown), or CHO cells were transfected with mGluR6-EGFP and labeled in nonpermeabilizing conditions with mGluR6 mAb 1438 to detect surface expression. EGFP fluorescence in the same field was imaged to detect total expression. Image processing conditions that permitted visualization of WT mGluR6 1438 labeling resulted in oversaturated images for some mutants. Images processed independently are shown in Fig. S5*B*. *Right*, quantification of surface/total expression, shown normalized to WT. Points represent means from independent experiments. *Inset*, CT mutants shown on an expanded y-axis. *C*, extracellular domains of ELFN1 and LRRTM4 were fused to Fc, and secreted protein was bound to protein G beads. Bound beads were then incubated with lysate of HEK 293F cells transfected with mGluR6. Input lysate, flow-through lysate, and bead-bound samples were blotted with anti-human antibody and mGluR6 mAb 312. *D*, *left*, example dose–response curves showing glutamate-induced G-protein activation. Error bars: means ± SEM of technical replicates. *Right*, efficacy and pEC50 values. Points represent independent experiments, and error bars show means ± SEM. ∗*p* ≤ 0.05; ∗∗*p* ≤ 0.01; ^#^*p* ≤ 0.001, one-way ANOVA with Dunnett’s posttest to compare all mutants to WT.

To examine the surface expression of mGluR6 mutants in heterologous cells, we exploited the extracellular epitope of clone 1438. Cells were transfected with mGluR6-EGFP and labeled in nonpermeabilizing conditions with 1438; EGFP fluorescence was imaged to measure total expression in the same cells (Fig. 10, *A* and *B*). The CT mutants had normal or partially reduced plasma membrane trafficking, though for the complete CT deletion Δ839–871 both surface expression and total expression were reduced, resulting in normal surface/total measurements. Surprisingly, the LBD mutants with large deletions had dramatically enhanced surface expression. All three smaller LBD deletions were similar to WT. In permeabilizing conditions, all constructs had similar labeling with 1438 (Fig. S5*A*), indicating that the enhanced surface expression seen with some mutants was not due to different accessibility of the mAb epitope. Attempting to use an N-terminal Myc tag following the predicted signal sequence (a.a. 1–23), instead of the native epitope, yielded different results, with LBD mutants apparently having reduced surface expression (Fig. S5*C*). However, labeling the same Myc-tagged constructs with 1438 recapitulated the enhanced surface expression seen with untagged proteins, indicating a problem with the Myc tag, possibly due to erroneous signal peptide cleavage, rather than changes in surface expression (Fig. S5*D*). These results suggest that caution is warranted when using N-terminal tags in proteins with N-terminal signal sequences. Removing the entire LBD (Δ24–511) surprisingly had no effect on apparent surface expression, detected with the Myc tag (the 1438 epitope is not present in this mutant) (Fig. S5*C*).

The surface expression results in heterologous cells show that the LBD deletion mutants are not grossly misfolded and are capable of normal, or even enhanced, plasma membrane trafficking. The fact that the same mutants are mislocalized in ON-BCs suggests that synaptic localization and plasma membrane trafficking are mediated by distinct mechanisms.

### Ligand-binding domain deletions abolish ELFN1 binding and G-protein activation

ELFN1 is a leucine-rich repeat protein that is expressed in rods and interacts trans-synaptically with mGluR6; in ELFN1 knockout mice, mGluR6 fails to accumulate at dendritic tips (21). To test the ability of mGluR6 LBD mutants to bind ELFN1, we performed pull-down experiments with the extracellular domain of ELFN1, or negative control protein LRRTM4, fused to Fc. Protein-G beads with bound Fc fusions were incubated with detergent-solubilized lysates of HEK cells expressing untagged mGluR6. In this assay, WT mGluR6, but none of the LBD mutants, bound to ELFN1 (Fig. 10*C*). All of the mGluR6 constructs were expressed at similar levels and migrated in SDS-PAGE at approximately the expected size of a dimer (Fig. 10*C*, left). The mutants were also present at similar levels in the flow-through (unbound fraction), indicating that the lack of binding observed was not due to degradation (Fig. 10*C*, middle). These results are consistent with a model in which ELFN1 binding is necessary for synaptic accumulation of mGluR6.

The ability of mGluR6 mutants to mediate glutamate-induced G-protein activation was tested using a Ca^2+^ mobilization assay in HEK cells. Cells were cotransfected with untagged mGluR6 and chimeric G_αqo_, which allows the normally G_o_-coupled mGluR6 to engage the G_q_ pathway for Ca^2+^ release from internal stores (28, 29). The LBD mutants were completely unable to mediate G-protein activation (Fig. 10*D*) despite normal or better surface expression (Fig. 10, *A* and *B*), probably due to impaired ligand binding. CT mutants Δ855–871 and Δ864–871 behaved similarly to WT in this assay, though the efficacy of Δ855-871 was slightly reduced. The complete CT deletion Δ839–871, in contrast, was completely nonfunctional; this may be due to the reduced absolute surface expression (Fig. 10*A*) and/or to a requirement for the CT in G-protein interaction. Since Δ839–871 was correctly localized to dendritic tips in BCs (Figs. 2 and 3), these results indicate that functional G-protein activation is not required for localization.

## Discussion

Deletion mutations within the LBD of mGluR6 revealed that multiple regions, or a tertiary structure requiring multiple regions, are required for dendritic tip localization in BCs (Fig. 2). These results could indicate the presence of a binding site for trafficking machinery or other BC proteins. However, all LBD mutants were also unable to bind to ELFN1 (Fig. 10), raising the possibility that ELFN1 binding is linked to dendritic tip localization. Indeed, in the Elfn1 knockout mouse, mGluR6 was mislocalized in rod BCs (21), though the rod synapses were also malformed at the ultrastructural level, which would likely cause secondary effects on protein localization. However, at cone synapses the related protein ELFN2 interacts with mGluR6, and double knockout of Elfn1 and Elfn2 in photoreceptors led to lack of mGluR6 at cone-ON BC dendritic tips without detectable structural defects (22). In hippocampal neurons, ELFN1 similarly mediated trans-synaptic recruitment of the related protein mGluR7 (30). These observations suggest a model in which a trans-synaptic interaction is required for synaptic accumulation of mGluR6. In this model, the protein could be either trafficked indiscriminately to the plasma membrane or specifically targeted to dendrite membranes; if trans-synaptic interaction fails to occur, mGluR6 may diffuse away in the plasma membrane or be recycled to an intracellular pool. Since all of the LBD mutants were competent for plasma membrane trafficking in HEK cells (Fig. 10), failure to bind ELFN1 could explain their mislocalization in BCs.

Though the LBD mutants were primarily intracellular in BCs (Fig. 6), it can be difficult to detect surface proteins without specifically labeling them by immunostaining in nonpermeabilizing conditions. All constructs including WT mGluR6 were primarily intracellular in heterologous cells as well, though the ability to label in nonpermeabilizing conditions allowed for detection of surface expression—all LBD mutants performed normally or better than WT in this assay (Figs. 10 and S5). The larger LBD deletions (Δ24–137 and Δ24–189) led to a dramatic (∼50 fold) enhancement of surface expression. This surprising result suggests some role for the LBD in regulating secretory trafficking. Efforts to recreate this phenotype with smaller deletions within the region 24 to 137 were not successful—all had surface expression similar to WT—suggesting that, like for dendritic tip localization and ELFN1 binding, a tertiary structure requiring multiple regions of the LBD is required. It is possible that secretory trafficking in BCs is regulated differently than in HEK and CHO cells. However, it is technically challenging to measure surface expression in retina tissue, making the heterologous cells a useful model. At the very least, these results indicate that the LBD mutants are capable of secretory pathway transit and plasma membrane insertion.

In SDS-PAGE of proteins expressed in HEK cells, all of the tested LBD mutants migrated at approximately double the expected size (Fig. 10), suggesting that all of them can form dimers. The mislocalization of LBD mutants in nob3 mice, identical to that observed in WT mice, indicates that either the mutants cannot heterodimerize with WT mGluR6 or that dimerization is not sufficient to drive localization. One possible explanation for this result is that complex formation with ELFN1 requires an interface containing two intact mGluR6 LBDs.

A previous report examining CT deletion mutants of mGluR6 found that some mutants had reduced surface expression in heterologous cells, suggesting the presence of a regulatory element in this region (31). We also observed reduced absolute surface expression of the CT mutant Δ839–871, though total expression was also reduced. In BCs, this mutant, as well as Δ855–871, had dendritic tip localization indistinguishable from that of WT mGluR6 (Fig. 2). However, the resolution of confocal microscopy is not sufficient to determine whether mGluR6 mutants are present in the postsynaptic membrane or in intracellular membranes in the dendritic tips. This localization pattern was maintained even in the absence of endogenous mGluR6 (Fig. 3), indicating that localization was not mediated by dimerization with a WT protein, and that the CT domain is dispensable for dendritic tip accumulation.

Mutations throughout the *GRM6* gene have been identified in CSNB patients, including missense mutations in the LBD, the cysteine-rich domain, and the transmembrane domain (18, 19, 32, 33, 34, 35, 36) (Figs. 1*A* and S6). All investigated missense mutant proteins were reported to have impaired plasma membrane trafficking in HEK cells (32), consistent with the loss of function CSNB phenotype. Though these results may appear contrary to our observations that even large deletions in the LBD do not impair surface expression, different mutations can have unpredictable effects on protein folding and function. An interesting future study would be to determine other phenotypes of CSNB mutants, including localization in BCs by electroporation.

As reported previously (16, 17), we observed TRPM1 mislocalization in the absence of mGluR6, though some TRPM1 was still detectable in dendritic tips (Fig. 4). Introduction of mGluR6-EGFP into the nob3 background was able to rescue this phenotype to some extent (Fig. 5). The mechanism of this functional interaction is unknown. Yeast two-hybrid experiments failed to demonstrate an interaction between mGluR6 and TRPM1 (37). However, further studies are needed to determine whether mGluR6 and TRPM1 can interact directly. The role of mGluR6 in TRPM1 localization does not necessarily imply a direct interaction. The postsynaptic LRR protein nyctalopin is also required for dendritic tip localization of TRPM1 (37), and interactions between TRPM1 and nyctalopin have been reported (16, 37). Synaptic localization of TRPM1 may require a multiprotein complex containing mGluR6, nyctalopin, and TRPM1, and possibly other proteins. One mechanism by which complex formation could mediate TRPM1 localization is by suppressing an ER retention signal and driving ER export. Numerous ion channel subunits utilize this method to achieve obligate multimerization (for example, (38, 39, 40, 41)). However, the observation that mGluR6 appears at dendritic tips before TRPM1 during development (42) argues against this model. Another possibility is that the presence of mGluR6 at the synapse could lead to triggering of TRPM1 plasma membrane insertion, or merely capturing and enriching small amounts of TRPM1 already present in the plasma membrane.

The CT mutant Δ855–871 mediated robust TRPM1 rescue, similar to WT (Fig. 5). In contrast, the complete CT deletion Δ839–871, although apparently mediating some rescue, did so to a significantly lesser extent. These results suggest that the mGluR6 CT, while dispensable for dendritic tip localization, does play a role in TRPM1 localization. The LBD mutants Δ24–58 and Δ59–111, which were mislocalized, were unsurprisingly also not able to mediate TRPM1 rescue.

In this study we tested synaptic localization of mGluR6 mutants *in vivo*. In theory, the ability of mutants to mediate synaptic transmission could also be assayed by introducing them into the nob3 background. However, transfection of BCs by electroporation of plasmid DNA is relatively inefficient and typically occurs in patches (23, 24, 43). While sometimes advantageous for imaging studies, the sparse transfection would likely make it challenging to observe phenotypes by electroretinogram recordings or behavioral experiments. Though partial rescue of ganglion cell responses and visual behavior in a photoreceptor degeneration model has been achieved by electroporation of light-activated channelrhodopsin in ON-BCs (43), most studies of functional rescue by expression of proteins in BCs have used recombinant AAV vectors (44, 45, 46, 47, 48, 49). In addition to photoreceptor degeneration models, rescue of CSNB models with ON-BC deficits has been attempted. Partial rescue in the spontaneous nyctalopin mutant Nyx^*nob*^ was achieved using AAV expressing nyctalopin in ON-BCs (48). However, AAV-mediated expression of mGluR6 in an mGluR6-null mouse failed to restore ON-BC function detectable by electroretinogram (49), possibly due to low expression or irreversible developmental defects. As a nonblinding disease, CSNB is not a priority candidate for clinical application of gene therapy. However, functional rescue experiments will be useful for investigating the postsynaptic signal transduction cascade in ON-BCs.

Dendritic tip localization of mGluR6 in ON-BCs is well established and has been observed with several polyclonal antibodies, all raised against CT peptides (5, 16, 50, 51, 52, 53). mGluR6 mAb 366, which binds to a CT epitope, also labeled dendritic tips exclusively. However, two novel mAbs with epitopes in the LBD and TM domains revealed the presence of an intracellular pool of mGluR6 in BC somas (Figs. 7 and 9). The intracellular mGluR6 is partially colocalized with the ER, similar to TRPM1 (27) (Fig. 8). These results highlight the fact that detection of native proteins can depend critically on fixation conditions and antibody epitope. The inability of CT epitope antibodies to detect the intracellular pool suggests that prior to delivery to dendritic tips, access to the CT may be hindered by complex formation. Since the CT was not required for dendritic tip localization of mGluR6 itself, this complex may be involved instead in bringing other proteins to the synapse, consistent with the impaired TRPM1 rescue ability of Δ839–871.

Besides its critically important role as a glutamate receptor, mGluR6 has several other functions in ON-BCs. It has multiple reported interaction partners—ELFN1/ELFN2 (21, 22, 54), nyctalopin (16, 37), and GPR179 (55); other unidentified proteins may be involved in trafficking intermediates. mGluR6 also appears to be centrally important for assembly of the postsynaptic signaling machinery, as mGluR6 is required for localization of several other proteins (16, 17, 55, 56), but is itself normally localized in the absence of other postsynaptic proteins (10, 57, 58, 59). Future studies will be important for identifying the structural basis of these interactions.

## Experimental procedures

### Expression constructs

pCDNA3.1 with mouse mGluR6 (NP_775548.2), cloned from retina cDNA, has been described (29). For surface expression measurements, constructs directing expression of a C-terminal EGFP fusion connected with a 7-a.a. flexible linker (mGluR6-GGGSGGG-EGFP) with or without a Myc tag inserted following the signal sequence (mGluR6[1–23]-Myc-mGluR6[24–871]-GGGSGGG-EGFP) were cloned into pCDNA3.1. Deletion mutants were made in both constructs, with N-terminal deletions starting immediately following either the signal sequence (mGluR6[1–23]) or the Myc tag. Signal peptide cleavage sites predicted using SignalP-5.0 (60) were between a.a. 23 and 24 for WT mGluR6 and all N-terminal mutants except Δ24–189, which had a predicted cleavage site between a.a. 21 and 22. For expression in ON-BCs, pGrm6P containing the *Grm6* promoter 200 bp critical region along with SV40 enhancer (61) was constructed from Addgene plasmid #18817 (a gift from Connie Cepko; RRID: Addgene_18817) as described (27). WT or mutant mGluR6-GGGSGGG-EGFP, or DsRed, was cloned into pGrm6P. pCDNA3.1 with the extracellular domain of mouse ELFN1 (a.a. 1–418) or mouse LRRTM4 (a.a. 1–425) fused to human Fc was described previously (24).

### Animals

All procedures were approved by the Baylor College of Medicine Animal Care and Use Committee. WT C57BL/6 mice were purchased from the Baylor College of Medicine Center for Comparative Medicine, *Grm6*^*nob3*^ mice (hereafter nob3) were purchased from Jackson Laboratory, and WT CD1 mice were from Charles River. To facilitate subretinal injections, nob3 mice were backcrossed to CD1 for five generations (Fig. S2). The region of genomic DNA containing the nob3 mutation (20) was PCR amplified from tail DNA with primers F (5′-TCCCTGAAAGCAGAGTACTGAAGG-3′) or F2 (5′-TGCTTGCCTTAACCCGTTCCGGTGC-3′) and R2 (5′-GTTCTAGGATGGGGTGAGTGTATC-3′). Sequencing of gel-purified PCR products with primer F2 revealed additional changes to the nucleotide sequence (Fig. S2, *A* and *C*) that permitted PCR genotyping using primers WT-F (5′-GAGCTCCCATCTCTTTTC-3′) and R2 to specifically amplify the WT allele, and nob3-F (5′-GAGCTCCCATCTCTTCTAT-3′) and R2 to specifically amplify the nob3 allele (Fig. S2*B*).

### Subretinal injection and electroporation

Subretinal injection and electroporation were performed at P0 essentially as described (23, 24, 62). Plasmid DNA was prepared using a Qiagen maxiprep kit and dissolved in water. pGrm6P-mGluR6-EGFP DNA (2 mg/ml) was supplemented with 10× PBS (final concentration 1×) and Fast Green dye (final concentration 0.1%) for injection. In most experiments pGrm6P-DsRed (1 mg/ml) was included to help identify transfected cells. The eyelid was opened and a pilot hole made with a 30G needle. A 33G blunt needle was positioned in the subretinal space and ∼450 nl of DNA solution was injected at 130 nl/s using a UMP3 Microsyringe Injector and Micro4 Controller (World Precision Instruments). Tweezer electrodes were placed across the eyes and five 50-ms 80 V pulses were delivered at 1-s intervals with an ECM 830 square wave electroporator (BTX Harvard Apparatus).

### Retina immunostaining

Injected animals were processed ∼4 to 6 weeks postinjection. Unless indicated otherwise, whole eyes were fixed in 2% PFA in PBS for 20 min, then washed extensively in PBS, and cryoprotected overnight at 4 °C in 30% sucrose in PBS. Corneas were removed, eyecups with lenses were embedded in OCT, and 10 to 12 μm sections were cut at −20 °C. Sections were blocked with blocking buffer (PBS with 10% donkey serum, 5% BSA, and 0.2% Triton X-100) for at least 2 h at RT, labeled overnight at 4 °C with primary antibodies followed by Alexa fluor-conjugated secondary antibodies (Invitrogen) 8 μg/ml for 2 h at RT, and mounted with Prolong Diamond (Invitrogen). Antibodies were diluted in blocking buffer.

### Cell transfection and immunostaining

HEK293 cells used were either purchased from the ATCC (#CRL-1573) or obtained from Michael Zhu (University of Texas); the latter were authenticated at the University of Texas MD Anderson Cancer Center Cytogenetics and Cell Authentication Core. HEK cells were maintained in DMEM (Corning) with 10% FBS (Sigma), HEK293 FreeStyle cells (HEK293F) (Gibco) were maintained in FreeStyle media (Gibco) supplemented with 2% FBS, and CHO cells were maintained in DMEM/F12 50/50 media (Corning) with 10% FBS. All cells were grown at 37 °C in a humidified incubator with 5% CO_2_. For transfection, cells were seeded on poly-D-lysine coated coverslips (HEK293, HEK293F) or uncoated coverslips (CHO) in 24-well plates and transfected with Lipofectamine 2000 (Invitrogen) and 0.6 μg of DNA according to the manufacturer instructions. Two days posttransfection, cells were fixed with 2% PFA in PBS for 10 min, washed in PBS, and blocked with PBSAT (PBS with 1% BSA and 0.1% Tween-20) or nonpermeabilizing PBSA (PBS with 1% BSA). Cells were labeled with Myc 9E10 or mGluR6 mAb 1438 for 1 h, followed by Alexa fluor-conjugated secondary antibodies (Invitrogen) 2 μg/ml for 30 min diluted in PBSAT or PBSA and mounted with Prolong Diamond.

### Primary antibodies

mGluR6: Generation of mGluR6 antibodies at the Baylor College of Medicine Protein and Monoclonal Antibody Production Core using full-length purified mouse mGluR6 was described previously (26), and clones 312 and 366 were validated with nob3 retina tissue in western blots (26) and IF (27) respectively. All clones are validated in this study. TRPM1: Clone 545H5 (26, 63) (isotype IgG2b) was previously validated with Trpm1 knockout retina tissue (26). Myc tag: c-Myc clone 9E10 hybridomas were obtained from the Developmental Studies Hybridoma Bank (The University of Iowa). mGluR6, TRPM1, and Myc hybridomas were cultured in Iscove’s Modification of DMEM (Corning) with either 15% FBS (Sigma) for expansion or 10% low IgG FBS (Sigma) for antibody purification. A column packed with protein G Sepharose Fast Flow (GE Healthcare) was equilibrated with wash buffer (20 mM sodium phosphate, pH 7) and loaded with culture supernatant supplemented with 50 mM Tris-Cl pH 8. The column was washed with wash buffer and eluted with 0.1 M glycine, pH 3. Eluates were immediately neutralized with 50 mM Tris pH 8, then buffer exchanged into PBS. Antibodies were stored at 4 °C in PBS with 0.02% Na Azide and used at the following concentrations: retina IF, 10 μg/ml; cell IF, 3 μg/ml; western, 1 μg/ml. Commercial antibodies: BiP/GRP78 (Abcam #ab21685, RRID: AB_2119834, 1 μg/ml), Na+/K+ ATPase (Santa Cruz Biotechnology #sc-28800, RRID: AB_2290063, 4 μg/ml), and Ribeye A-domain (Synaptic Systems #192-103, RRID: AB_2086775, 1 μg/ml).

### Confocal microscopy and image processing

Confocal images were acquired with a Zeiss LSM-710 microscope and 63× oil immersion objective (Zeiss, Plan-Apochromat 63×/1.4 Oil DIC M27). Alexa 647, Alexa 555, and EGFP/Alexa 488 were detected sequentially with 633 nm, 561 nm, and 488 nm lasers. For display of images showing localization, input levels were adjusted differently for different images to highlight the EGFP signal. For display of images showing rescue of TRPM1 puncta or mGluR6 surface expression in heterologous cells, images in each panel were adjusted identically. Single optical sections are shown unless indicated otherwise. Quantifications were performed with raw images processed as follows.

For quantification of OPL puncta localization in retina, maximum projections of z-stacks (∼8–14 images, 1024 × 512 pixels, z-interval 0.5 μm, x-y resolution 52.7 nm/pixel, 8 bit) were analyzed. All images were acquired with identical settings. First, the inner nuclear layer was manually removed in Photoshop (Adobe) to create a masked image containing the OPL puncta. The remainder of the analysis was performed in Mathematica v.12 (Wolfram). The TRPM1 channels in both original full-field and masked images were scaled to the minimum and maximum of the full-field image, then a background value estimated as the mean of the first row pixel intensity was subtracted for both channels. OPL puncta were detected in the masked background-subtracted image using MorphologicalComponents with method ConvexHull and threshold values in TRPM1 and EGFP channels of 0.3 and 0.15, respectively. Components with ≥150 pixels were removed, and the total intensity of the remaining puncta divided by the total intensity of the full-field background-subtracted image was reported. For analysis of colocalization, line profiles were obtained from single optical slices in ImageJ, using a 4-px wide line and the Plot Profile tool. Profiles were normalized to minimum and maximum values.

For quantification of TRPM1 localization rescue in retina, single images (1024 × 512 pixels, 65.9 nm/pixel, 8 bit) acquired with identical settings were analyzed. Images were masked to remove the INL, and TRPM1 channel scaled to minimum and maximum as above. OPL puncta were detected as above except background subtraction was omitted, threshold values in both TRPM1 and EGFP channels were 0.15, and components ≥100 pixels were removed. Total intensity of the remaining puncta in the TRPM1 channel was reported. For puncta-forming mGluR6 constructs (WT, Δ855–871, and Δ839–871) images were programmatically designated as an untransfected region if the total intensity of components in the EGFP channel was <2. For non-puncta-forming mGluR6 mutants (Δ24–58, Δ59–111), images were manually designated as untransfected regions if no EGFP signal was evident.

For quantification of surface expression in heterologous cells, images (1024 × 1024 pixels, 132 nm/pixel, 8 bit) were analyzed. For each experiment, WT and mutant images were acquired together with identical settings. Images were thresholded at 2% to approximate background subtraction, and total intensity of the 1438 channel (surface) divided by EGFP (total) was measured. Values for four images for each construct were averaged and normalized to WT from the same day. In experiments with CHO cells, the transfection efficiency was quite low, and an additional step was used to omit background signal from untransfected cells: masks were created by binarizing the EGFP channel with threshold of 5%, and the remaining intensity after applying the mask in both channels was measured.

### ELFN1 pull-down assay

HEK293 or HEK293F cells were seeded in 6-well plates. Wells to be transfected with Fc fusion constructs were changed to media with low IgG serum and transfected with Lipofectamine 2000 and 4 μg pCDNA3.1 containing ELFN1(NT)-Fc or LRRTM4(NT)-Fc. Approximately 24 h later, other wells were transfected with 4 μg pCDNA3.1 with untagged WT or mutant mGluR6. Approximately 40 h later, media from wells transfected with Fc fusions was harvested, centrifuged to remove cells, and supplemented with 1/20 volume Tris pH 7.4 and a dash of PMSF. Protein G agarose beads (Pierce) (100 μl) were added and allowed to bind for ∼3 h with end-over-end mixing. Beads were washed with PBS and divided into six tubes for pull-down reactions. Cells transfected with mGluR6 constructs were washed with PBS, resuspended in 1 ml of lysis buffer (PBS with 1% Triton X-100, 50 mM supplemental NaCl, and ∼1.6× Complete protease inhibitors (Roche)), and incubated on ice for 15 min. Lysates were centrifuged in a microcentrifuge at 5000 rpm for 10 min at 4 °C, and supernatant was added to tubes containing Fc fusion-protein G beads. Reactions were incubated at 4 °C with end-over-end mixing for 90 min, and beads were washed four times with lysis buffer.

Samples of input lysate, flow-through after binding, and 5× equivalent amount of beads were resolved by SDS-PAGE and blotted with mGluR6 mAb 312 (1 μg/ml) followed by anti-mouse-kappa light chain conjugated to HRP (Jackson) (1:10,000) or with anti-human-IgG conjugated to IRDye 800CW (Licor) (1:5000) to detect Fc fusion proteins. Blots were imaged with a digital imager (Azure).

### Calcium mobilization assay

HEK293 cells were seeded in black clear-bottom poly-D-lysine coated 96-well plates (∼45,000 cells/well). The next day, cells were transfected with 50 ng/well pCDNA3.1 containing untagged WT or mutant mGluR6 along with 150 ng/well pCDNA3.1 containing chimeric G_αqo_ (28, 64). Approximately 36 to 40 h later, wells were washed with KRH buffer (120 mM NaCl, 4.7 mM KCl, 2.2 mM CaCl_2_, 10 mM HEPES, 1.2 mM KH_2_PO_4_, 1.2 mM MgSO_4_, 1.8 g/l glucose, pH 7.4, supplemented with 1 mM probenecid) and incubated with 2.7 μM Fluo-4 AM (Invitrogen) in KRH with 0.01% Pluronic F-127 (Biotium) for 1 h at room temperature in the dark. Cells were washed again with KRH, then 100 μl KRH was added to each well. Assays were performed at 37 °C in a Flex Station 3 (Molecular Devices) with cell plate and drugs prewarmed to 37 °C. Fluorescence measurements were acquired from the bottom of the plate (Ex/Em 488/520); 50 μl 3× L-Glutamate diluted in KRH was added after 20 s. Each mutant was assayed in triplicate on three different days. Raw data were baseline corrected by subtracting the average of the first ∼20 s, then dose–response curves were constructed from the average of the maximum value and the two flanking points for each trace. Triplicate values were fit with sigmoidal dose response curves in Prism v.9 (GraphPad). Control wells with no drug were included in the curve fits by assigning them a drug concentration of 10^−15^ M. Log(EC50) values were determined from curve fits. Relative efficacy values were calculated from data normalized to the top plateau of WT from the same plate. Plateaus of curve fits were used, except for mutants with no response to ligand, the maximum of averaged technical replicates was used instead.

### Experimental design and statistical analyses

Quantification of images for localization was performed with at least three images each from at least three animals, except for some mislocalized mutants with identical phenotypes, two animals were used. Quantification of images for TRPM1 rescue was performed with at least three images each from transfected and untransfected regions (technical replicates for each animal are shown in Fig. S3), from at least four animals. Quantification of surface expression in transfected cells was performed with four images each from at least three independent experiments. In some cases results from different cell types were combined, as indicated in the figure. Individual biological/independent replicates are shown in all plots unless indicated otherwise. For calcium mobilization assays, example dose–response curves that are representative of three independent experiments are shown; measurements were derived from three independent experiments. For ELFN1 pull-down experiments, western blots shown are representative of three independent experiments. Images presented for qualitative interpretation only (not used for quantification) are representative of images acquired from 2 to 3 animals. Statistical analyses were performed in Prism v.5 (GraphPad). For comparison of multiple mutants to WT, one-way ANOVA with Dunnett’s posttest was used. Comparisons of genotypes, or of transfected and untransfected regions, were performed with two-tailed paired or unpaired *t*-tests as described in figure legends.

## Data availability

The data that support the findings of this study are available by request from the corresponding author.

## Supporting information

This article contains supporting information (20, 36, 65, 66, 67).

## Conflict of interest

The authors declare that they have no conflicts of interest with the contents of this article.
