# Supplementary material for: The mGluR6 ligand-binding domain, but not the C-terminal domain, is required for synaptic localization in retinal ON-bipolar cells
Source: J Biol Chem. 2021 Nov 15;297(6):101418. doi: 10.1016/j.jbc.2021.101418 (PMC8671642; doi:10.1016/j.jbc.2021.101418)
Supplement: Supplemental Figures S1–S6 and Table S1 [file mmc1.pdf]

Supporting Information

The mGluR6 ligand-binding domain, but not the C-terminal domain, is required for synaptic localization in retinal ON-bipolar cells

Melina A. Agosto[1], Abiodun Adefola R. Adeosun [1,2], Nitin Kumar[1], Theodore G. Wensel[1,2]

[1] Verna and Marrs McLean Department of Biochemistry and Molecular Biology and
[2] Pharmacology and Chemical Biology Graduate Program
Baylor College of Medicine, Houston TX, USA

Supplemental Figure S1. Example of puncta detection method using the same images shown in Figure 2.

Supplemental Figure S2. Back-crossing of nob3 mice with CD1.

Supplemental Figure S3. Technical replicates for dendritic tip quantification shown in Figures 4 and 5.

Supplemental Figure S4. Initial mAb screening results including clones not shown in Figure 7.

Supplemental Figure S5. Surface expression of mGluR6 mutants.

Supplemental Figure S6. CSNB mutations.

Supplemental Table S1. Summary of mGluR6 mAb properties.

Supplemental Figure S1

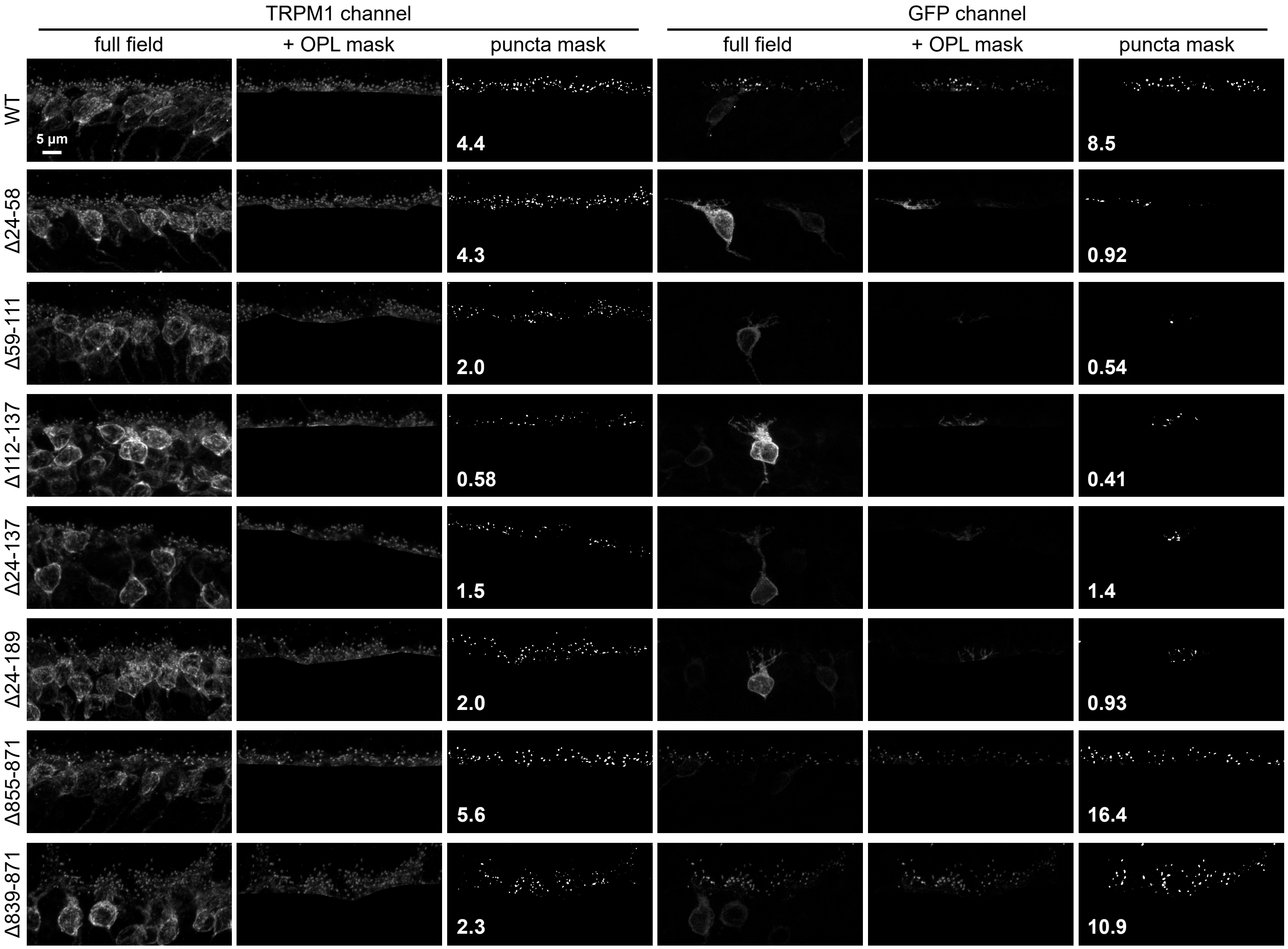

Figure S1. Example of puncta detection method using the same images shown in Figure 2. Masks were drawn manually to remove the bottom portion of images containing the INL and keep the OPL (OPL mask). Puncta were then automatically detected using MorphologicalComponents in Mathematica as described in Methods (puncta mask). Numbers indicate the intensity of puncta (determined after applying the puncta mask) as a percentage of the total EGFP intensity, for the images shown. For LBD mutants, some false positive puncta were detected, evidenced by lack of colocalization with TRPM1 in Figure 2.

Supplemental Figure S2

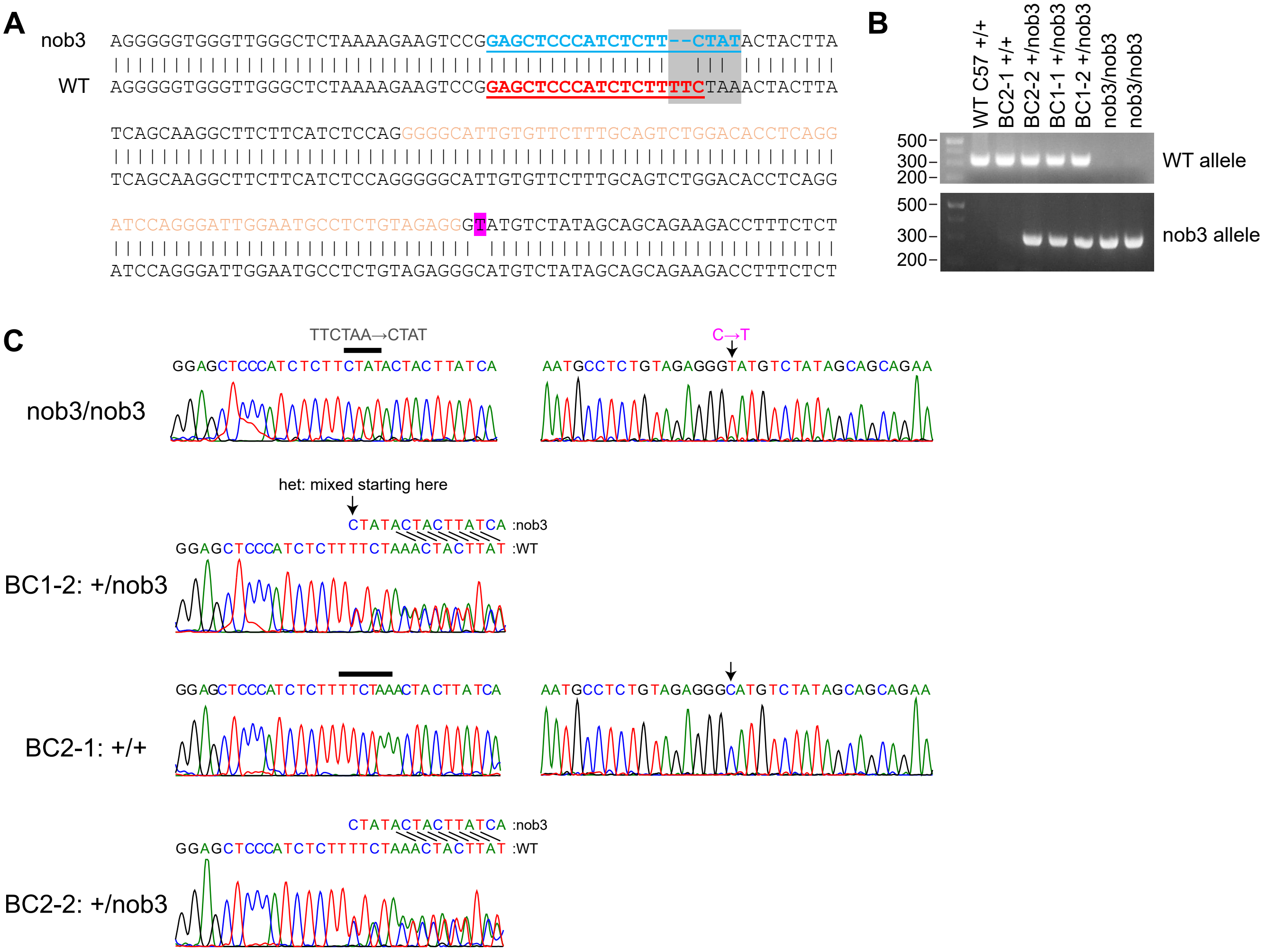

Figure S2. Back-crossing of nob3 mice with CD1. (A) Sequencing of the nob3 allele identified an additional mutation (TTCTAA→CTAT, grey) immediately upstream of the reported C→T mutation (Maddox et al. 2008) (magenta). This additional mutation permitted genotyping using PCR primers specifically detecting the nob3 allele (nob3-F, blue) or the WT allele (WT-F, red). The spurious exon included in the nob3 transcript is shown in peach. **(B)** Example genotyping results for back-cross 1 (BC1) and back-cross 2 (BC2) animals using primers WT-F or nob3-F and a common reverse primer. **(C)** Sequence verification of some animals shown in (B).

Supplemental Figure S3

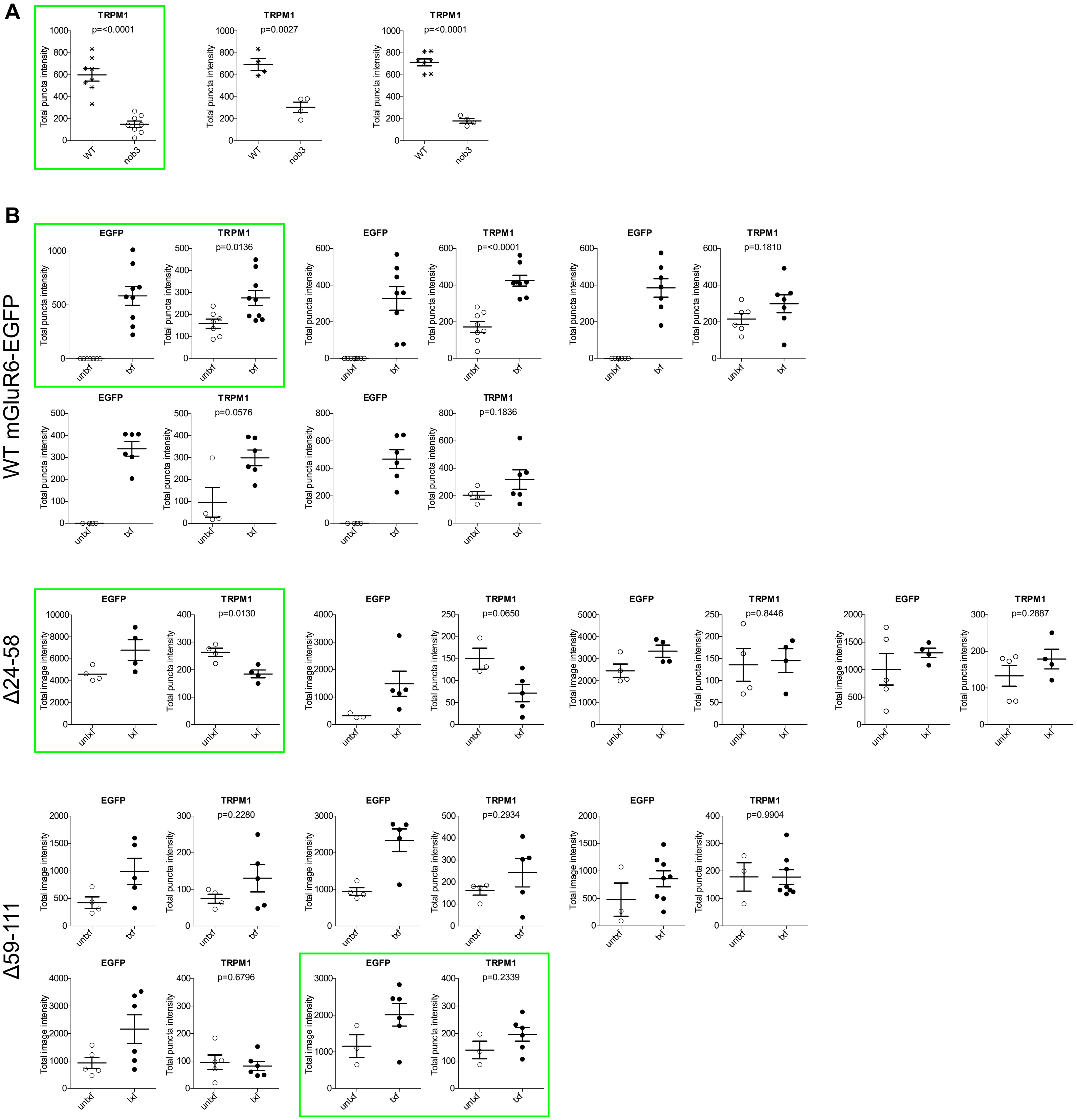

Supplemental Figure S3 (cont'd)

B (cont'd)

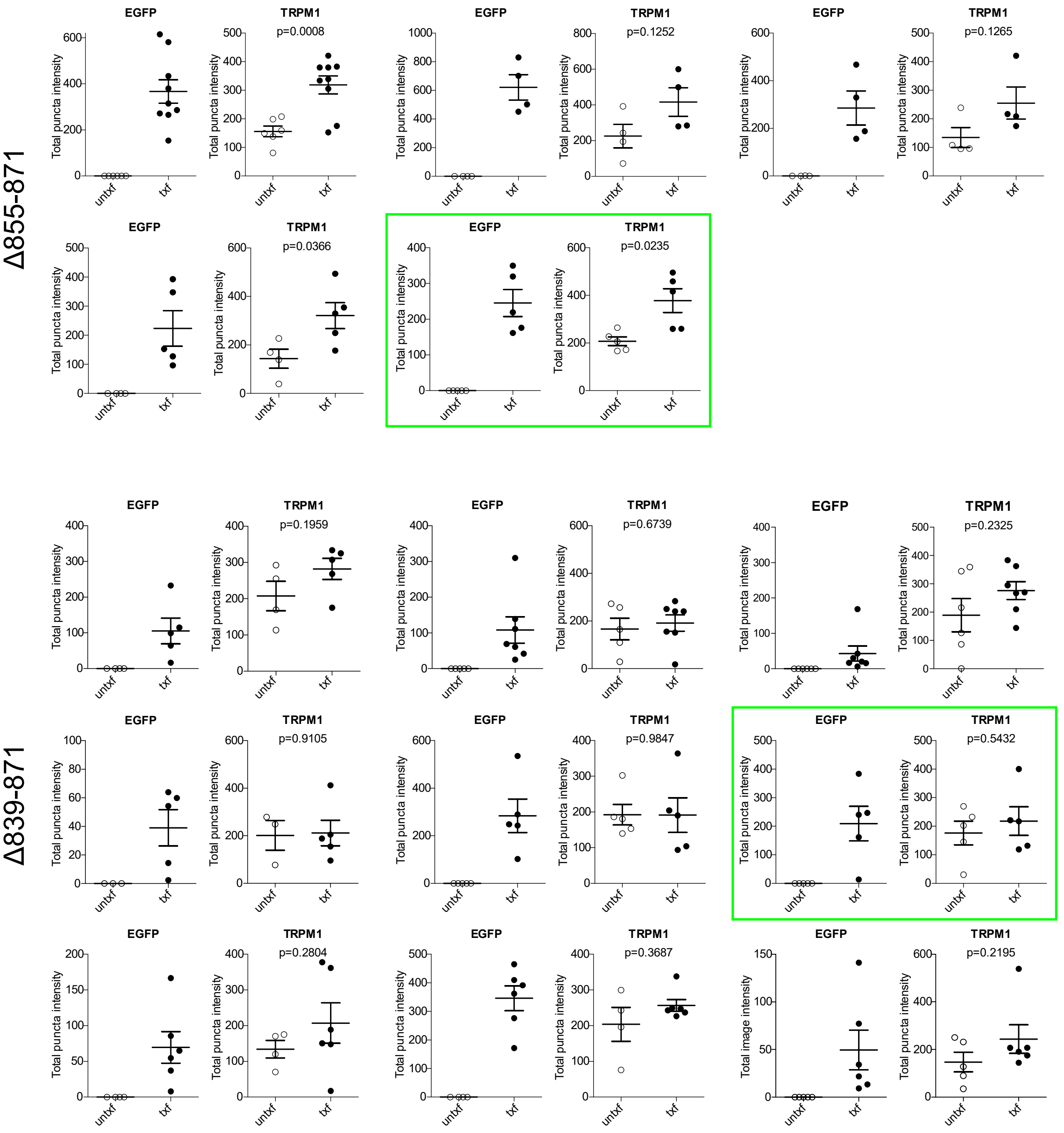

Figure S3. Technical replicates for dendritic tip quantification shown in Figures 4 and 5. (A) Reduced dendritic tip TRPM1 in CD1/nob3 mice. Quantification of technical replicates are shown; each plot represents a different animal. **(B)** Rescue of TRPM1 dendritic tip localization by electroporated mGluR6-EGFP. Each pair of plots shows quantification of technical replicate images from one animal. Untransfected (untxf) and transfected (txf) regions were assigned as described in Methods, and TRPM1 OPL puncta intensity compared with two-tailed unpaired t-tests. Examples used in main figures are outlined in green.

Supplemental Figure S4

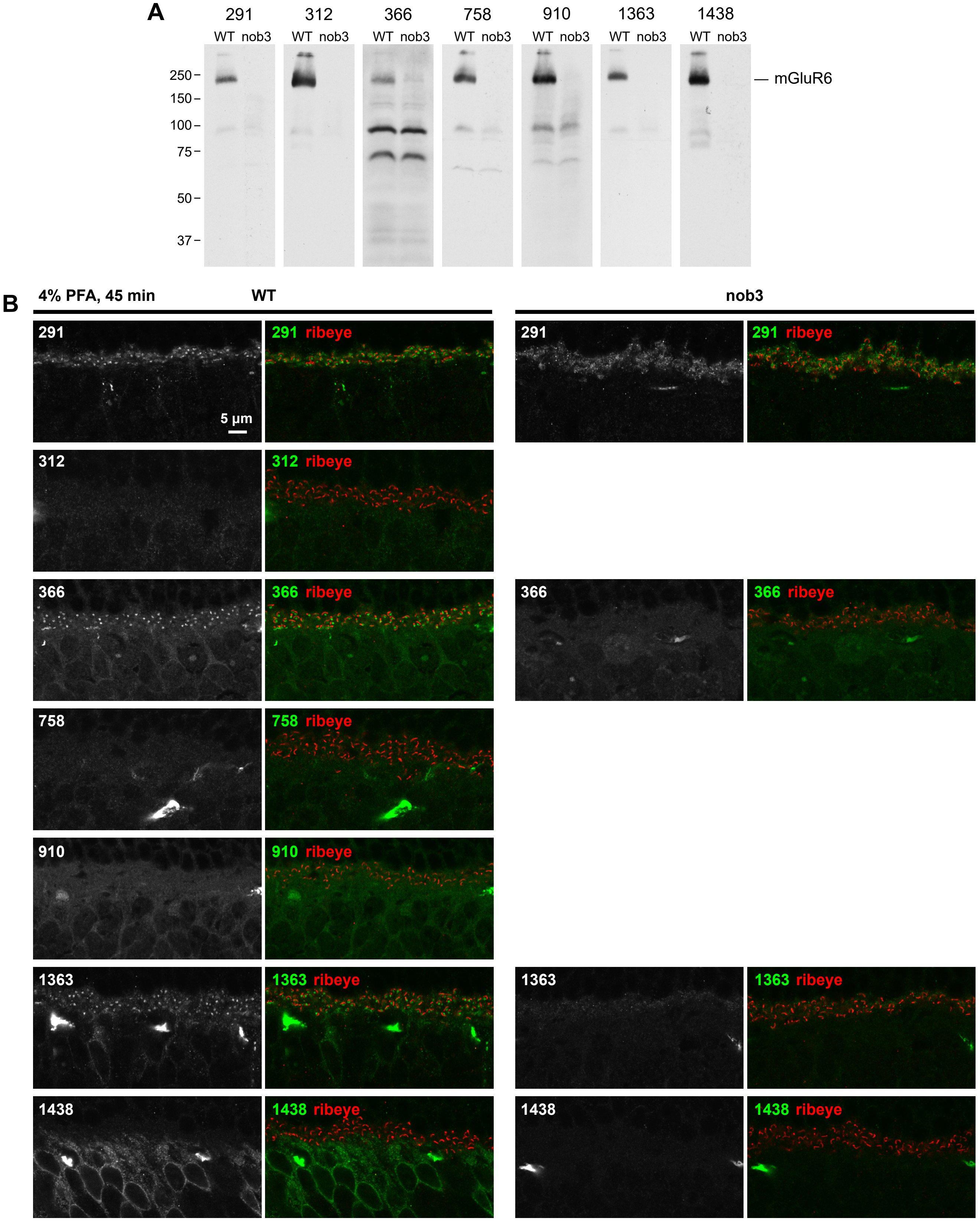

Figure S4. Initial mAb screening results including clones not shown in Figure 7. (A) WT and nob3 whole retina lysate (~0.5 retina/lane) were blotted with undiluted hybridoma culture supernatant, followed by HRP-conjugated anti-mouse antibody. **(B)** WT and nob3 retina sections were labeled with undiluted hybridoma culture supernatant.

Supplemental Figure S5

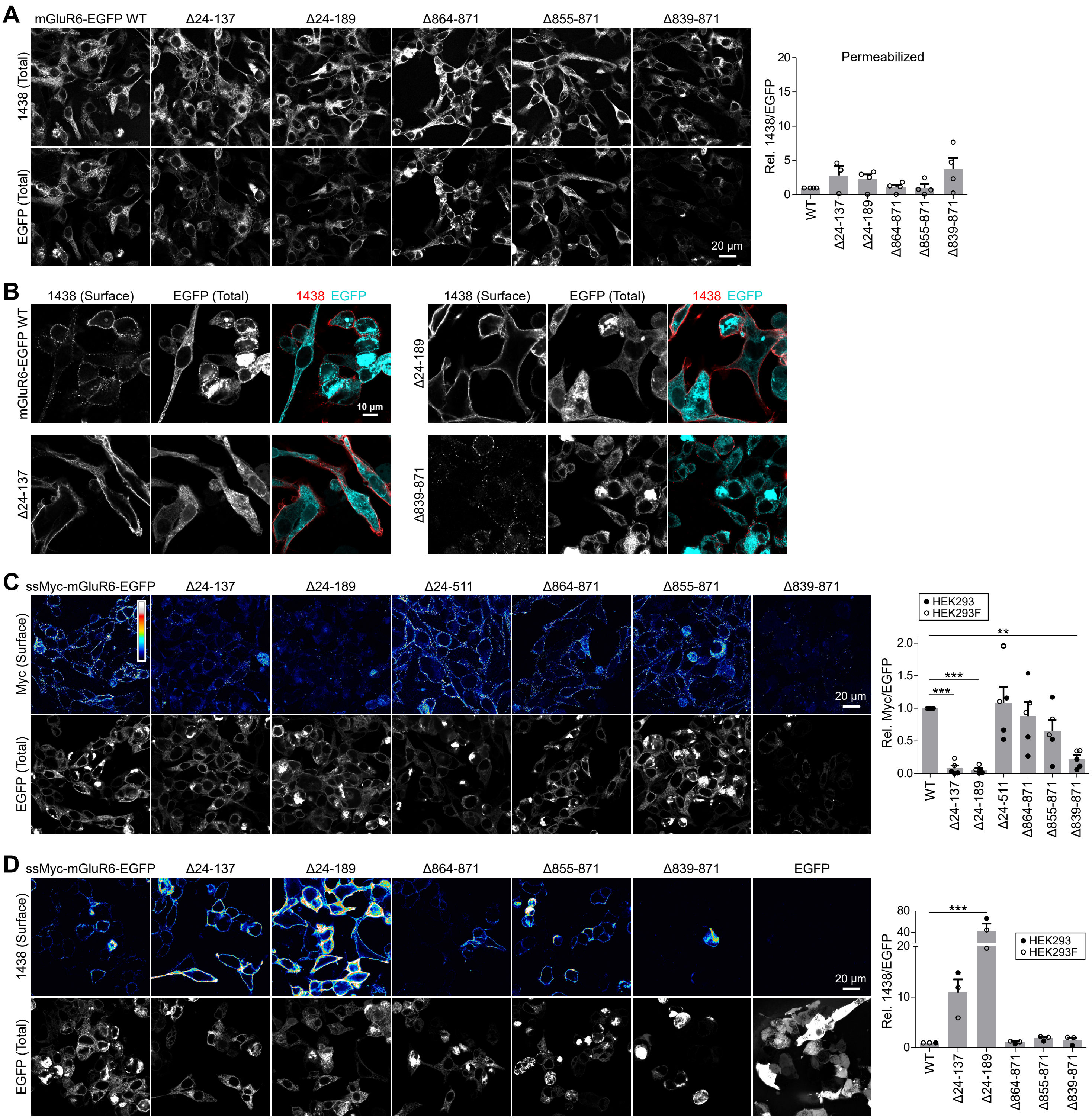

Figure S5. Surface expression of mGluR6 mutants. (A) HEK 293F cells transfected with mGluR6-EGFP were labeled in permeabilizing conditions with mGluR6 mAb 1438. **(B)** Magnified views from images in Figure 10A, with image processing conditions adjusted independently for each construct. Labeling intensity cannot be compared in these images. **(C,D)** HEK 293 or HEK 293F cells transfected with ss-Myc-mGluR6-EGFP were labeled in non-permeabilizing conditions with Myc antibody (C) or mGluR6 mAb 1438 (D).

Supplemental Figure S6

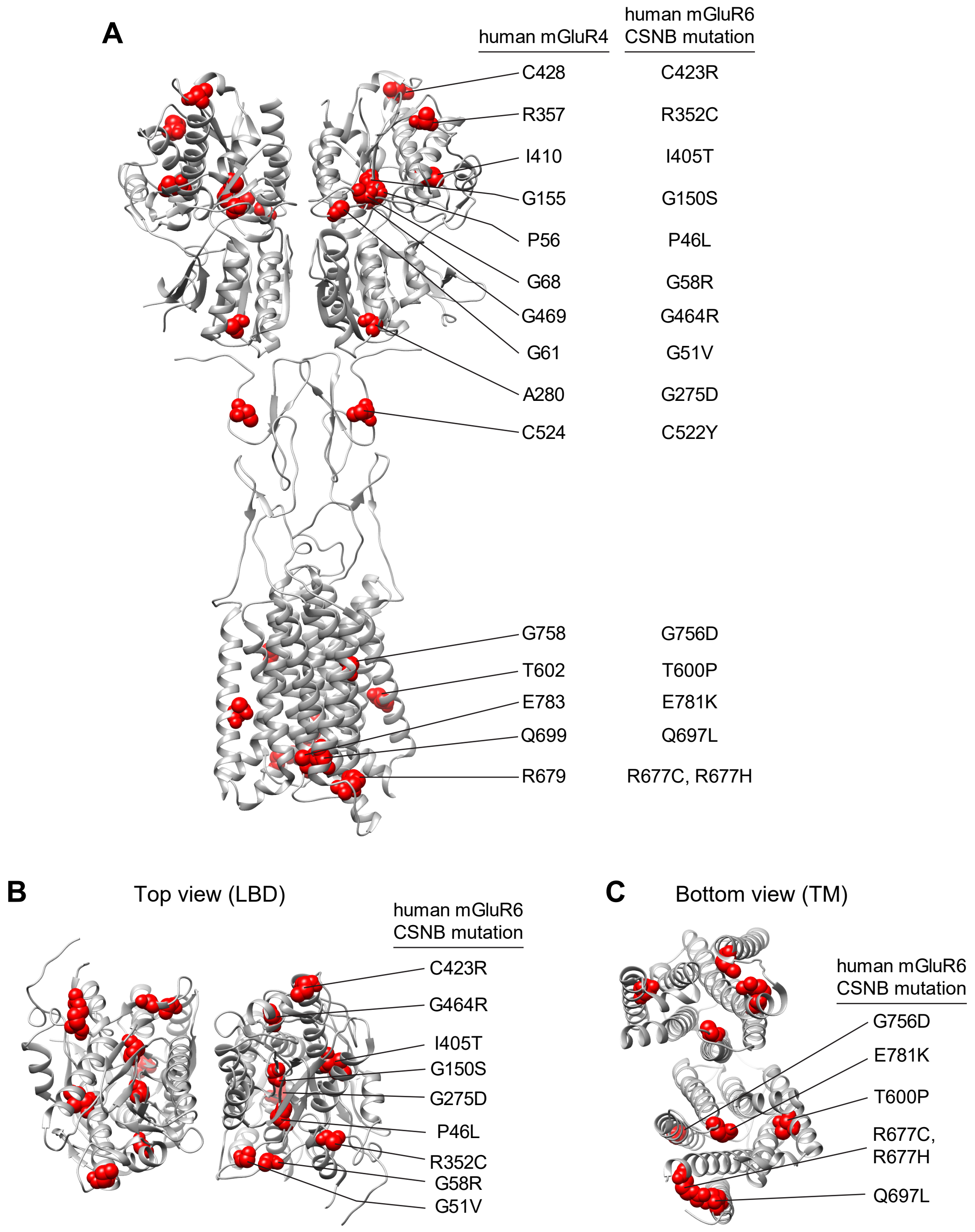

Figure S6. CSNB mutations. Homologous positions of CSNB missense mutations are shown as red spheres on the structure of human mGluR4 (PDB 7E9H, Lin et al. 2021). Side view **(A)**, top view of the LBD **(B)**, and bottom view of the TM domain **(C)** are shown. CSNB mutations are from Zeitz et al. 2015, Liu et al. 2019, and Tourville et al. 2019.

Supplemental Table S1. Summary of mGluR6 mAb properties.

Clone	Retina: WB	Retina: IF (harsh fix)[a,b]	Retina: IF (mild fix)[c]	HEK: extracellular	Epitope[d]
758	yes	no	n.d.	n.d.	LBD - N5a
1438	yes	soma	dt	yes	LBD - N5b
312	yes	no	no	n.d.	LBD - N7c
910	yes	no	no	n.d.	LBD/CRD - N7d
291	yes	dt + nonspecific OPL	dt + nonspecific OPL	no	EL2
1363	yes	dt + soma	dt	no	EL2
366	yes	dt	dt	no	CT

[a] Eyes were fixed with 4% PFA for 45 min
[b] dt, dendritic tips; OPL, outer plexiform layer
[c] Eyes were fixed with 2% PFA for 10 min
[d] LBD, ligand-binding domain; CRD, cysteine-rich domain; EL2, extracellular loop 2; CT, C-terminal domain
